# Early impact of donor *CYP3A5* genotype and Graft-to-Recipient Weight Ratio on tacrolimus pharmacokinetics in pediatric liver transplant patients

**DOI:** 10.1038/s41598-020-79574-7

**Published:** 2021-01-11

**Authors:** Michele Pinon, Amedeo De Nicolò, Antonio Pizzol, Miriam Antonucci, Antonio D’Avolio, Loredana Serpe, Dominic Dell’Olio, Silvia Catalano, Francesco Tandoi, Renato Romagnoli, Roberto Canaparo, Pier Luigi Calvo

**Affiliations:** 1 Pediatric Gastroenterology Unit, Regina Margherita Children’s Hospital, AOU Città della Salute e della Scienza di Torino, University of Turin, Turin, Italy; 2 Unit of Infectious Diseases, Department of Medical Sciences, University of Turin, Amedeo di Savoia Hospital, Turin, Italy; 3 Department of Drug Science and Technology, University of Turin, Turin, Italy; 4 Regional Transplant Center, AOU Città della Salute e della Scienza di Torino, Turin, Italy; 5 General Surgery, Liver Transplant Center, AOU Città della Salute e della Scienza di Torino, University of Turin, Turin, Italy

**Keywords:** Liver, Liver, Genetic testing, Clinical pharmacology, Pharmacogenetics, Pharmacokinetics

## Abstract

Tacrolimus (TAC) pharmacokinetics is influenced by the donor *CYP3A5* genotype and the age of pediatric liver recipients. However, an optimization of a genotype-based algorithm for determining TAC starting is needed to earlier achieve stable target levels. As the graft itself is responsible for its metabolism, the Graft-to-Recipient Weight Ratio (GRWR) might play a role in TAC dose requirements. A single-center study was carried out in a cohort of 49 pediatric recipients to analyse the impact of patient and graft characteristics on TAC pharmacokinetics during the first 15 post-transplant days. Children < 2 years received grafts with a significantly higher GRWR (4.2%) than children between 2–8 (2.6%) and over 8 (2.7%). TAC concentration/weight-adjusted dose ratio was significantly lower in recipients from CYP3A5*1/*3 donors or with extra-large (GRWR > 5%) or large (GRWR 3–5%) grafts. The donor *CYP3A5* genotype and GRWR were the only significant predictors of the TAC weight adjusted doses. Patients with a GRWR > 4% had a higher risk of acute rejection, observed in 20/49 (41%) patients. In conclusion, TAC starting dose could be guided according to the donor *CYP3A5* genotype and GRWR, allowing for a quicker achievement of target concentrations and eventually reducing the risk of rejection.

## Introduction

The introduction of the calcineurin inhibitor drug tacrolimus (TAC) has improved patient survival in pediatric liver transplant patients. Nevertheless, the best way of employing this drug is still a matter of intense debate^1,2^, since TAC is characterized by a narrow therapeutic index, a high inter- and intra-individual pharmacokinetic, pharmacodynamic and pharmacogenetic variability and by significant adverse effects^3^.

Therefore, TAC therapeutic drug monitoring (TDM) in whole blood is essential to optimize clinical outcomes and minimize adverse effects. Nevertheless, the use of the TDM results for dose adjustment is time consuming before a stable and optimum TAC blood concentration can be reached and may lead to over or under-dosing determining drug toxicity or acute rejection. Therefore, better strategies for TDM are required to establish the optimal TAC dosage, especially in the early post-transplant period^4^.

Orally administered TAC, a lipophilic drug, has a variable absorption, first pass metabolism and limited bioavailability^3^. *CYP3A4* and *CYP3A5* are the primary enzymes responsible for TAC metabolism in the liver and to a lesser extent in enterocytes^5^. It is already well known that CYP3A5*3 allele, that is found in 90% of the Caucasian population, determines a lower TAC catabolism, whilst the *1 allele is related to a fast metabolism and, consequently, to a relatively lower TAC exposure^6^: non-expresser patients are poor metabolizers, have higher TAC blood concentrations and should be given the standard recommended starting dose, whereas individuals carrying at least one CYP3A5*1 allele are intermediate or extensive metabolizers and require a higher tacrolimus dose. The role of *CYP3A5* genotype was established by a systematic review carried out on 596 pediatric transplant recipients^7^. In the most recent consensus report on TAC personalized therapy, *CYP3A5* genotype is indicated as the main pharmacogenetic biomarker to be used in clinical practice for determination of the initial dose of tacrolimus in order to rapidly reach a therapeutic concentration^2^. Particularly, the donor *CYP3A5* genotype was reported to influence TAC pharmacokinetics in liver transplant patients, whereas the recipient *CYP3A5* genotype had no impact on TAC blood concentration^8–12^. Beyond *CYP3A5*, the determination of CYP3A4*22 genotype could explain the residual variability in TAC pharmacokinetics^3^. Lastly, *ABCB1* (encoding P-glycoprotein, P-gp) polymorphisms seem to be less relevant in TAC pharmacokinetics^13^.

Apart from the genetic make-up, clinical and graft factors may affect TAC concentrations, e.g. patient age and gender, transplant type, baseline renal and liver function, concomitant use of corticosteroids or other drugs, serum albumin concentration and hematocrit, food administration or diarrhoea^14^. Among these, the recipient age has been pointed out as a main factor influencing TAC concentrations: younger pediatric patients require higher TAC doses than do older patients or adults to achieve similar TAC concentrations, probably due to a wider volume of distribution or a faster drug metabolism^12,15,16^.

In this regard, a sliding scale algorithm for a genotype guided TAC starting dose, stratified by the recipient age, was designed by Min et al., with the lowest dose in older (> 6 years) *CYP3A5* non-expressers and the highest dose in younger (≤ 6 years) *CYP3A5* expressers. This genotype-guided dosing was compared to a standard dosing in a cohort of pediatric solid organ transplant patients^17^, obtaining an earlier attainment of target levels and stable therapeutic concentrations with significantly fewer out-of-range results.

Indeed, TAC clearance could be dependent on the graft size, as the graft itself is responsible for its metabolism. This implies that, theoretically, the best marker of graft size could well be the Graft-to-Recipient Weight Ratio (GRWR), indicating the normalized size of the graft based on the patient’s weight. GRWR remains the most commonly used parameter for decision making in transplant surgery to calculate the optimal graft size for the recipient, so far due to its simplicity and practicality for clinical application. GRWR should be within the range 1% to 3% to avoid major complications related to size mismatching between the graft and the recipient, which may result in graft failure or even patient death if not appropriately handled^18^. GRWR has recently been correlated to TAC pharmacokinetics in a large Japanese cohort of pediatric living donor liver transplants^19^.

A further refinement and optimization of a genotype-based algorithm for determining TAC starting dose would be of advantage, in order to reach an earlier achievement of stable target immunosuppression levels and avoid adverse effects.

We present here our single-center study designed to evaluate the influence genetic polymorphisms and other patient and graft factors have onTAC blood levels in a cohort of pediatric liver transplant patients in the first two post-transplant weeks.

## Materials and methods

### Patient enrolment and data collection

All pediatric patients who consecutively underwent liver transplantation from January 2007 to December 2014 at *AOU Città della Salute e della Scienza di Torino*, Italy, were assessed for eligibility. This study is an extended pharmacokinetic and pharmacogenetic analysis on a previously published study by our group, which was limited to the first post-transplant day^12^ and did not take graft weight into account.

Inclusion criteria were as follows: (a) age < 18 years old at listing; (b) oral TAC immunosuppressive monotherapy and (c) informed consent of legal guardian. Exclusion criteria were as follows: (a) contra indications to oral tacrolimus; (b) multiple organ transplants or retransplants; and (c) declined consent.

Donor and recipient DNA samples were provided by the Regional Transplantation Center (CRT Piemonte e Valle d'Aosta, Italy), extracted after enrollment and analyzed.

Donor, recipient and graft characteristics, along with laboratory data, were collected from the medical records.

For the analysis, the recipients were divided into groups according to their age: under 2 years of age, between 2 and 8 and older than 8 years. GRWR was calculated using the formula graft weight/recipient weight and it was expressed as a percentage. The liver transplants were categorized into the following three groups according to the GRWR:

- small-for-size graft, if GRWR was < 1%;
- size-matched graft, if GRWR was between 1 and 3%;
- large-for-size graft, if GRWR was between 3 and 5%;
- extra-large-for-size graft, if GRWR was > 5%.

The study was approved by the Institutional Review Board (Comitato Etico Interaziendale *AOU Città della Salutee della Scienza di Torino*, Italy) and written informed consent was obtained from all patients’ parents. The study was carried out in compliance with the provisions of the Declaration of Helsinki and the Good Clinical Practice guidelines. No organs from executed prisoners were used.

### Immunosuppression protocol

The recipients received basiliximab at transplant (day 0) and on day 4 and/or steroids as induction. All patients were postoperatively administered oral TAC as capsules; naso-gastric administration was used in few cases.

The initial target TAC dose was 0.075 mg/Kg every 12 h, subsequently adjusted according to TAC whole blood concentrations (C_0_). The target C_0_ for the first 2 weeks was set between 8 and 12 ng/mL, in line with our center’s protocol and in agreement with other study protocols.

Any suspicion of acute rejection was assessed by liver biopsy. All histologically proven episodes of acute rejection were treated by increasing the TAC dose and/or a bolus dose of steroids.

### Tacrolimus pharmacokinetic analysis

TAC daily doses (mg/Kg/die), trough TAC blood concentrations (ng/mL) and the concentration/weight-adjusted dose ratio (C/D/Kg) were determined daily during the first 2 post-liver transplantation weeks. C/D/Kg was used as a marker of TAC bioavailability to avoid any confounding effect due to the different TAC pro-kilo doses and dose variations during treatment.

Blood sampling was performed at the end of the inter-dose interval (just before the new drug intake), with sodium/EDTA Vacutainer tubes. The TAC concentrations were determined by the Masstrak Immunosuppressants XE kit (Chromsystems, Gräfelfing, Germany), based on a UltraPerformance Liquid Chromatography Tandem Mass Spectrometry (UPLC-MS/MS) method.

### Genotype identification

Donor and recipient DNA samples were genotyped for CYP3A5*3, CYP3A4*22, *ABCB1 3435C* > *T* and *1199A* > *G* using the polymerase chain reaction (PCR) with TaqMan (Thermo Fisher Scientific). The other most common polymorphisms of *ABCB1*, i.e. 2677G > T and *1236 C* > *T*, were not analyzed, since they are generally reported to be in linkage disequilibrium with *3435C* > *T* polymorphism^14^.

### Statistical analysis

Data were analyzed by SPSS 24.0 software package (IBM, Armonk, NY). Categorical variables were summarized as counts and percentages and continuous variables as means + /- SD or medians and inter-quartiles ranges. Differences between groups in the continuous variables were determined by the Mann–Whitney or Kruskal–Wallis tests when more than one group was involved. The Fisher exact test or χ^2^-square test were used for categorical data. Univariate and multivariate linear regression analysis were performed to test the predictive value of variables showing significant correlation with TAC concentrations. Absence of significant collinearity of covariates and significant correlation with the dependent variables were considered as preliminary assumptions for regression analysis. In case of collinearity, only the variable with the highest predictive value by univariate regression was included in the multivariate analysis. All the tests were two tailed and a *p *value of ≤ 0.05 was considered statistically significant. The confidence intervals (CI) were calculated at the 95% level. ROC curve was used to identify cut-off values.

## Results

### Patient and graft characteristics

A total of 49 pediatric patients on TAC immunosuppressive monotherapy were included in this study.

Patient and graft characteristics are summarized in Table 1. All liver transplants were from deceased donors. Twenty-five recipients were under 2 years of age (51%), 16 were between 2 and 8 (32.7%) and 8 (16.3%) were older than 8 years old. The most frequent indications for liver transplant were biliary atresia, 26 pts (53.1%), hepatoblastoma, 12 pts (24.5%) and sclerosing cholangitis, 5 pts (10.2%). Other causes of end-stage liver disease were: Alagille syndrome, 1 pt (2%), progressive familial intrahepatic cholestasis, 3 pts (6.2%), cystic fibrosis, 1 pt (2%), and urea cycle disorder, 1 pt (2%). Induction immunosuppressive therapy was administered as follows: basiliximab in 33/49 (67%), basiliximab and steroids in 7/49 (14%) and steroids in 9/49 (19%).

**Table 1:** Patients baseline features at the date of transplantation and graft characteristics. GRWR, Graft-to-Recipient Weight Ratio.

		Recipients	Donors
Age at transplant, yrs, median (IQR)		2.3 (0.3–17.3)	12 (0.6–47)
Gender, N (%)		Males 23 (47%)	Males 29 (59%)
		Females 26(53%)	Females 20 (41%)
Ethnicity, N (%)		Caucasian 40 (82%)	–
		North African 3 (6%)	
		Sub-Saharan 2 (4%)	
		American 3 (6%)	
		Asian 1 (2%)	
Body weight, median (IQR)		12.8 kg (7.4–21.1)	–
ALT, median (IQR)		Day 1: 774 U/L (320–1518)	–
		Day 7: 111 U/L (IQR 75–246)	
		Day 15: 67 U/L (IQR 46–115)	
INR, median (IQR)		Day 1: 1.94 (1.73–2.62)	–
		Day 7: 1.22 (1.14–1.32)	
		Day 15: 1.20 (1.06–1.34)	
Graft Type, N (%)		Whole liver 20 (41%)	–
		Split liver 23 (47%)	
		Reduced 6 (12%)	
GRWR, median (IQR)		3.3% (2.5–4.4)	–
Gene polymorphism		N (%)	N (%)
CYP3A5*3	*1/*3	7 (14.3%)	14 (28.6%)
	*3/*3	42 (85.7%)	35 (71.4%)
CYP3A4*22	*1/*1	47 (95.9%)	42 (85.7%)
	*1/*22	2 (4.1%)	7 (14.3%)
*ABCB1 3435 C* > *T*	*C/C*	17 (34.7%)	18 (36.7%)
	*C/T*	26 (53.1%)	25 (51.0%)
	*T/T*	6 (12.2%)	6 (12.2%)
*ABCB1 1199 G* > *A*	*G/G*	42 (85.7%)	41 (83.7%)
	*G/A*	7 (14.3%)	8 (16.3%)

The *CYP3A5*, *CYP3A4* and *ABCB1* genotype frequencies did not deviate from the Hardy–Weinberg equilibrium. Genotype frequency was not significantly different between transplant recipients and donors and showed no association with age or gender. The total allelic frequency was similar to that observed in other populations made up in great majority of Caucasian subjects^1,20–22^.

Patients’ weight and graft weight resulted significantly correlated (r = 0.815, r^2^ 0.664, *p* value < 0.001), indicating a not perfectly proportional graft matching.

The median GRWR was 3.3% (IQR 2.5–4.4). Among all the enrolled patients, respectively 43%, 41% and 16% showed a GRWR between 1 and 3% (normal), 3% and 5% (large) or more than 5% (extra-large). No small-for-size grafts (GRWR < 1%) were transplanted.

GRWR was statistically significantly correlated with the recipient age (r = 0.479, r^2^ = 0.229, *p *value < 0.001). Median GRWRs were 4.2% (IQR 3.45–5.17), 2.6% (IQR 2.01–4.13) and 2.7% (IQR 1.59–2.98) for patients’ age groups < 2 years old, between 2 and 8 and > 8 years old, respectively (*p *value = 0.002). Children under 2 years of age received liver grafts with a higher GRWR than children between 2–8 (*p *value = 0.011) and over 8 (*p *value = 0.001). There was no difference in the GRWR values between children 2–8 years old and those over 8 years (*p *value = 0.417).

Whole, reduced and split livers respectively represented 45%, 5% and 50% of size-matched grafts (GRWR 1–3%), 16%, 16% and 68% of large grafts (GRWR 3–5%) and 62.5%, 12.5%, and 25% of extra-large grafts (GRWR > 5%).

Median values of ALT values were significantly higher in patients who received large and extra-large grafts. There was a statistically significant correlation between ALT and GRWR on the 1st post-transplant day (r^2^ = 0.155;*p *value = 0.043), thereafter this correlation did not reach statistical significance.

### Tacrolimus pharmacokinetics and patient characteristics

**Figure 1:**
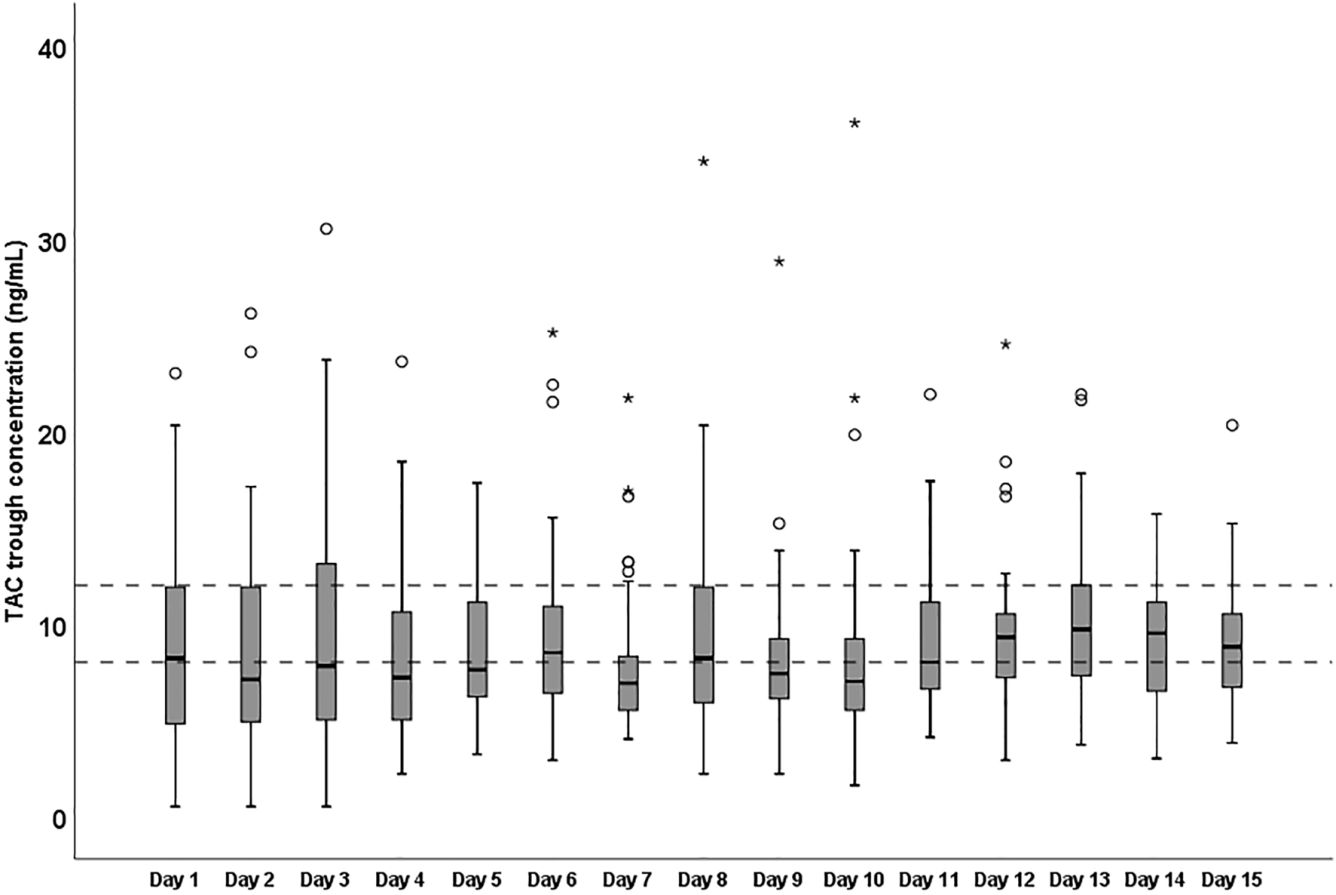
Daily TAC blood concentration after liver transplant. TAC, tacrolimus.

Figure 1 shows the TAC whole blood concentrations during the first 15 post-transplant days. There was a gradual increase in the number of patients who achieved the target C_0_ level (8–12 ng/mL) during this period, most likely due to TDM-guided dose adjustments, i.e. the percentage of patients who achieved target therapeutic ranges at day 1 and 15 increased from 23.6% to 42.9%.

In the first 7 days of treatment, there was a significant correlation with the initial (day 1) dose corrected by graft weight (r between 0.316 and 0.480, *p *values between 0.008 and 0.044 in the range of 2–8 days), with the dose corrected by the patients’ weight (r between 0.247 and 0.402, *p *values between 0.001 and 0.019) and with the graft weight (r between − 0.328 and − 0.389, *p *values between 0.006 and 0.023 in the range of 4–6 days). These data suggest that graft weight may affect the achievement of the desired TAC concentrations.

Attention was then focused on the factors affecting the inter-individual variability in the TAC C/D/Kg. Female recipients showed significantly higher C/D/Kg on the 1st post-transplant day (*p *value = 0.03) than male patients. TAC C/D/Kg was significantly lower in children under 8 years of age, particularly in those under 2, from the 1st post-transplant day to the 7th (Table 2). Those patients who received livers from adult (*p *value = 0.046) and female donors (*p *value = 0.048) had a significantly higher TAC C/D/Kg on the 1st post-transplant day and on the 3rd and 4th post-transplant days, respectively.

**Table 2:** TAC concentration/weight-adjusted dose ratio (C/D/Kg) in the first 15 days from transplantation stratified by donor CYP3A5 genotype, recipient age and GRWR. Asterisks (*) indicate statistically significant differences between the marked group and the others.

Post-transplant days		TAC C/D/Kg (median and interquartile range)														
		Day 1	Day 2	Day 3	Day 4	Day 5	Day 6	Day 7	Day 8	Day 9	Day 10	Day 11	Day 12	Day 13	Day 14	Day 15
**Donor CYP3A5 gen**	**Quartiles**															
CYP3A5 *1/*3	1	32.5	27.9	33.6	28.0	27.1	23.3	24.5	21.5	20.4	17.2	17.0	21.0	23.0	20.6	19.6
	2	62.4	51.5	47.5	42.4	41.5	57.3	40.7	39.7	27.5	28.4	34.0	34.1	34.2	30.4	32.7
	3	93.1	74.0	63.4	73.5	80.5	85.5	59.8	68.0	44.7	45.1	50.5	65.3	113.3	65.2	136.3
CYP3A5*3/*3	1	71.5	53.1	61.4	46.0	45.7	38.1	35.3	40.3	33.7	29.7	28.2	29.6	37.7	30.9	35.1
	2	147.3*	85.8*	122.2*	111.7*	93.0*	74.0*	67.2*	70.2*	60.8*	57.6*	54.6	63.0	60.1	54.7	50.4
	3	194.3	144.4	199.3	190.5	164.4	156.2	121.9	103.0	123.8	94.2	84.3	103.7	92.9	81.8	84.0
*p *values	< 0.001	< 0.001	< 0.001	< 0.001	< 0.001	0.050	0.022	0.023	< 0.001	0.025	0.071	0.122	0.124	0.073	0.272	
**Recipientage**	**Quartiles**															
< 2 years	1	55.7	30.8	33.6	35.3	31.2	22.1	24.5	24.0	20.9	18.1	20.5	20.6	27.8	24.2	18.4
	2	94.8	62.8	58.7	49.0	45.1	57.0	39.0	43.2	34.4	35.6	30.0	39.4	46.1	32.8	36.9
	3	172.36	94.3	187.1	165.2	96.0	93.5	73.8	93.3	70.5	86.2	72.6	120.5	93.5	79.8	78.4
2–8 years	1	39.60	29.7	22.3	29.6	40.8	33.6	25.4	38.1	24.0	22.6	26.8	23.5	27.5	27.8	31.9
	2	70.0	56.3	66.9	57.0	70.4	60.2	57.2	62.7	41.9	41.9	44.7	46.3	43.3	47.3	53.6
	3	102.86	85.6	120.2	127.0	128.6	109.4	92.4	96.8	78.5	76.2	81.2	76.8	89.5	63.6	77.8
≥ 8 years	1	117.0	83.0	91.3	63.9	64.3	49.5	40.2	45.2	34.0	40.5	39.7	38.5	40.6	48.0	41.5
	2	138.9*	128.4*	137.8*	125.4*	137.9*	136.0*	94.3*	76.1	67.4	58.8	62.5	66.4	68.5	77.3	82.0
	3	205.0	166.2	198.1	244.4	183.8	177.0	141.9	109.4	125.1	92.5	85.3	93.0	128.5	128.0	127.4
*p *values	0.022	0.002	0.036	0.056	0.016	0.024	0.024	0.200	0.073	0.208	0.210	0.648	0.552	0.051	0.108	
**GRWR**	**Quartiles**															
Normal (2–3%)	1	63.8	52.6	47.8	45.5	40.3	37.4	36.4	39.7	30.6	35.1	45.9	36.2	28.1	35.1	32.5
	2	92.7	74.1	87.1	64.8	94.6	95.7	61.3	65.8	60.8	57.0	62.5	61.3	55.0	56.0	74.3
	3°	168.1	138.0	196.4	187.9	157.3	156.2	123.5	81.9	117.0	83.1	79.0	79.7	96.0	97.0	85.9
Large (3–5%)	1°	65.5	55.4	61.7	41.2	37.4	44.6	26.1	30.9	23.8	17.0	21.4	26.0	37.5	27.7	17.2
	2°	153.1	80.9	143.9	108.4	64.7	59.5	41.3	43.3	36.8	33.8	37.2	46.4	67.2	58.2	55.1
	3°	203.8	143.4	189.2	169.8	96.0	78.3	79.7	77.9	87.5	95.4	88.6	144.2	93.5	82.9	106.1
Extra-large (> 5%)	1°	32.8	30.8	20.8	19.6	32.5	21.9	22.3	25.9	21.9	17.4	17.4	20.3	24.4	21.8	18.9
	2°	56.1	53.1	38.9*	37.0*	45.1*	30.4*	25.4*	43.3*	28.7*	32.6	25.9	27.7	39.5	29.1	36.2
	3°	161.4	71.8	53.5	76.3	99.2	49.1	51.5	110.7	71.8	82.4	71.4	58.8	50.1	32.9	56.4
*p *values	0.348	0.142	0.015	0.013	0.002	< 0.001	< 0.001	0.023	0.008	0.092	0.845	0.594	0.833	0.017	0.210	

The levels of ALT were not correlated to TAC C/D/Kg, whereas a higher value of PT-INR on the 1st and the 5th post-transplant days correlated to a higher TAC C/D/Kg in the time-lapse between the 2nd and the 4th day (*p *values between 0.002 and 0.010) and between the 10th and the 15th day (*p *values < 0.01), respectively. Corticosteroids administered as induction therapy did not influence TAC C/D/Kg.

When both donor and recipient genotypes were taken into consideration, it was observed that the TAC C/D/Kg were significantly lower in patients who received a liver from CYP3A5*1/*3 donors compared to those who were recipients from donors homozygous for the *3 allele. This difference was statistically significant from the first post-transplant day through to the 10th (Table 2). Conversely, the recipient *CYP3A5* genotype had no significant influence on TAC levels in the early post-transplant period. Recipients receiving organs from *1/*3 donors had significantly higher TAC dose requirements than recipients from *3/*3 donors, regardless of the recipients’ genotype. Neither the donor nor recipient *CYP3A4* or *ABCB1* polymorphisms influenced significantly the TAC dose requirements in the first 15 post-transplant days. TAC C/D/Kg tended to be higher in *3435 C/T* than in *3435 C/C* recipients, in *3435 T/T* than in *3435 C/T* patients and in *1199 G/A* than in *1199 G/G* recipients, although these differences did not reach the statistical significance.

The type of transplanted graft did not influence the TAC blood levels, whereas TAC C/D/Kg was significantly lower in extra-large or large grafts from the 3rd to the 9th post-transplant day (Table 2).

The TAC C/D/Kg data were stratified by the main parameters showing a statistically significant association over a longer time range (recipients’ age, donor *CYP3A5* genotype and GRWR), as reported in Table 2.

### Predictive factors of tacrolimus pharmacokinetics

Univariate and a multivariate linear regression analysis were performed to identify the most relevant independent factors affecting TAC C/D/Kg. The univariate analysis confirmed the results from the correlation tests. Factors which showed at least one significant predictive power on TAC C/D/Kg during the first 15 post-transplant days included the recipient age, the donor gender and *CYP3A5* genotype, the GRWR and the PT-INR.

The multivariate analysis documented that the recipient age, the donor *CYP3A5* genotype and gender were independent predictors of TAC C/D/Kg on the 1st post-transplant day, whereas only the recipient age, the donor *CYP3A5* genotype and PT-INR at day 1 remained statistically significant on the 2nd day. The recipient age and the PT-INR at day 1 were no longer significant predictors from the 3rd and the 4th day respectively, whereas from the 3rd day the GRWR started to be a significant predictor of TAC C/D/Kg. The donor CYP3A5 genotype and GRWR remained significant predictors of the TAC C/D/Kg up until the 10th post-transplant day, when PT-INR at day 5 remained the only predictor of TAC C/D/Kg (Table 3).

**Table 3:** Overview of the statistical significance of the predictive value of patients and graft characteristics for TAC concentration normalized by weight-adjusted dose (C/D/Kg). *p *values were calculated by multivariate linear regression analysis: only variables showing at least one significant *p *value by univariate analysis were included in the multivariate testing. NS = not significant and excluded from the model. CYP3A5 *1/*3 was coded as 1 while *3/*3 was coded as 2. *GRWR* was coded as percentage.

Days	TAC C/D/Kg (*p *values)														
	1°	2°	3°	4°	5°	6°	7°	8°	9°	10°	11°	12°	13°	14°	15°
Regression R^2^	0.301	0.329	0.384	0.296	0.187	0.236	0.361	0.185	0.173	0.36	0.243	0.179	0.187	0.195	0.205
F statistic (d.f., res)	9.49	7.7	8.07	8.4	4.49	6.2	11.03	3.82	3.75	4.38	8.7	5.27	6.48	8.74	9.01
(d.f., res.) to put together with F statistic, as marked above	(2, 46)	(3, 45)	(3, 45)	(2,46)	(2, 46)	(2, 46)	(2, 46)	(2, 46)	(2, 46)	(2, 46)	(1, 47)	(1, 47)	(1, 47)	(1, 47)	(1, 47)
Donor *CYP3A5* (Unstand. B coef.)	< 0.001	0.002	< 0.001	< 0.001	0.014	0.034	0.002	0.02	0.024	0.024	ns	ns	ns	ns	ns
(Unstand. B coef.) to put together with DONOR CYP3A5, as marked above	-9.2	− 75	− 107.4	− 87.3	− 40.6	− 60.1	− 43.4	− 35.2	− 45.4	− 12.7					
GRWR (Unstand. B coef.)	0.558	0.326	0.01 (− 21.6)	0.039 (− 15.9)	0.022 (− 11.7)	0.004 (− 27.2)	0.001 (− 16.2)	0.027 (− 17.3)	0.045 (− 15.2)	ns	ns	ns	ns	ns	ns
Recipient age (Unstand. B coef.)	0.001 (4.1)	0.013 (2.8)	ns	ns	ns	ns	ns	ns	ns	ns	ns	ns	ns	ns	ns
Recipient gender	ns	ns	ns	ns	ns	ns	ns	ns	ns	ns	ns	ns	ns	ns	ns
Donor age	ns	ns	ns	ns	ns	ns	ns	ns	ns	ns	ns	ns	ns	ns	ns
Donor gender (Unstand. B coef.)	0.008 (− 10.2)	ns	ns	ns	ns	ns	ns	ns	ns	ns	ns	ns	ns	ns	ns
INR day 1 (Unstand. B coef.)	ns	0.040 (− 35.5)	0.045 (5.1)	ns	ns	ns	ns	ns	ns	ns	ns	ns	ns	ns	ns
INR day 5 (Unstand. B coef.)	ns	ns	ns	ns	ns	ns	ns	ns	Ns	0.042 (130.7)	0.001-(95.2)	0.012 (100.2)	0.005 (105.6)	0.001 (110.2)	< 0.001 (111.3)
Intercept	26.5	40	7.9	13.7	57.1	80.1	43.4	36.2	31.4	− 125	− 105	− 60.7	− 68.2	− 70.3	− 71.2

**Figure 2:**
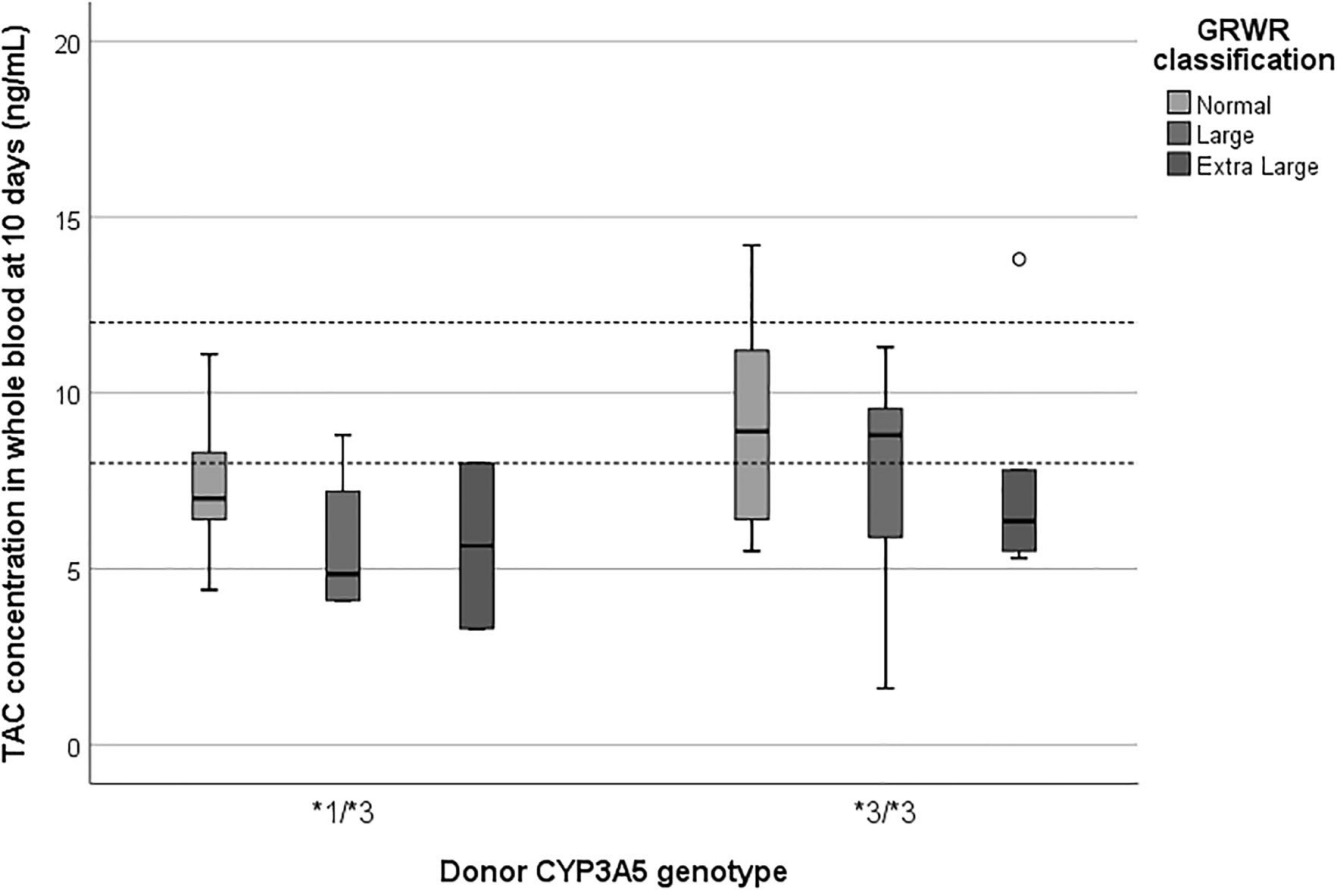
TAC blood concentrations on the 10th post-transplant day stratified by GRWR and the donor CYP3A5 genotype. TAC, tacrolimus; GRWR, Graft-to-Recipient Weight Ratio.

The influence GRWR has on TAC pharmacokinetics according to the donor CYP3A5 polymorphism was analyzed. Recipients from *1/*3 donors rarely achieved TAC concentrations within the therapeutic range, whereas only patients with extra-large grafts did not reach adequate target TAC concentrations among recipients from *3/*3 donors. Indeed, patients who received an extra-large-for-size graft required higher TAC doses than recipients with a large or size-matched graft regardless the donor *CYP3A5* genotype. Figure 2 shows the impact of GRWR on TAC blood concentrations stratified by the donor *CYP3A5* genotype on the 10th post-transplant day. Nevertheless, the differences in TAC concentrations between GRWR groups resulted not statistically significant (*p *value = 0.095), probably due to the low sample size and the variability in the administered dose between groups.

### Graft size correlation with tacrolimus kinetics and the weight-adjusted dose required

A linear regression analysis was performed between the GRWR and the weight-adjusted dose during treatment, to estimate the impact graft size has on the need for dose adjustments during treatment. Noteworthy is that the GRWR was a statistically significant predictor of the weight adjusted dose between the 10th and the 15th, indicating a predictive value on the required TAC dose. *CYP3A5* genotype also resulted to be an independent predictor of the weight-adjusted dose of TAC (Table 4).

**Table 4:** Summary of the distribution of the required TAC weight-adjusted doses, after stratification on *CYP3A5* genotype and GRWR classes. CYP3A5 *1/*3 was coded as 1 while *3/*3 was coded as 2. GRWR was coded as percentage.

Donor CYP3A5 genotype	GRWR			TAC weight-adjusted dose (mg/Kg/day)															
				Day 0	Day 1	Day 2	Day 3	Day 4	Day 5	Day 6	Day 7	Day 8	Day 9	Day 10	Day 11	Day 12	Day 13	Day 14	Day 15
*1/*3	Normal	N		8	8	8	8	8	8	8	8	8	8	8	8	8	8	8	8
		Quartiles	1°	0.036	0.1	0.102	0.131	0.159	0.147	0.113	0.152	0.2	0.207	0	0	0.156	0.14	0.166	0.166
			2°	0.052	0.137	0.138	0.194	0.189	0.198	0.169	0.185	0.216	0.241	0.158	0.14	0.25	0.3	0.3	0.3
			3°	0.109	0.246	0.294	0.266	0.224	0.256	0.224	0.354	0.258	0.306	0.263	0.262	0.298	0.323	0.323	0.323
	Large	N	4	4	4	4	4	4	4	4	4	4	4	4	4	4	4	4	
		Quartiles	1°	0.039	0.046	0.069	0.098	0.116	0.116	0.105	0.123	0.138	0.173	0.203	0.267	0.12	0.12	0.143	0.143
			2°	0.053	0.073	0.098	0.18	0.16	0.176	0.137	0.144	0.177	0.216	0.236	0.274	0.267	0.267	0.32	0.32
			3°	0.117	0.22	0.18	0.25	0.25	0.308	0.188	0.188	0.25	0.31	0.31	0.32	0.32	0.32	0.384	0.384
	Extra-large	N	2	2	2	2	2	2	2	2	2	2	2	2	2	2	2	2	
		Quartiles	1°	0.067	0.067	0.133	0.133	0.089	0.089	0.089	0.089	0.089	0.179	0.089	0.089	0.089	0.467	0.179	0.179
			2°	0.078	0.123	0.156	0.156	0.145	0.2	0.167	0.211	0.133	0.256	0.278	0.278	0.278	0.467	0.323	0.533
			3°	-	-	-	-	-	-	-	-	-	-	-	-	-	-	-	-
*3/*3	Normal	N	11	11	11	11	11	11	11	11	11	11	11	11	11	11	11	11	
		Quartiles	1°	0.045	0.05	0.052	0.045	0.05	0.05	0.05	0.061	0.05	0.05	0	0.094	0.051	0.076	0.105	0.105
			2°	0.052	0.089	0.075	0.063	0.053	0.061	0.067	0.098	0.1	0.105	0.121	0.129	0.118	0.111	0.145	0.143
			3°	0.105	0.133	0.115	0.11	0.1	0.085	0.121	0.138	0.134	0.138	0.134	0.159	0.139	0.143	0.167	0.157
	Large	N		17	17	17	17	17	17	17	17	17	17	17	17	17	17	17	17
		Quartiles	1°	0.053	0.057	0.058	0.058	0.078	0.073	0.059	0.086	0.097	0.08	0.07	0.072	0.06	0.058	0.072	0.078
			2°	0.06	0.085	0.064	0.065	0.091	0.118	0.136	0.126	0.188	0.195	0.174	0.179	0.122	0.126	0.187	0.16
			3°	0.08	0.145	0.083	0.081	0.132	0.218	0.197	0.221	0.282	0.312	0.379	0.311	0.235	0.274	0.268	0.369
	Extra-large	N	7	7	7	7	7	7	7	7	7	7	7	7	7	7	7	7	
		Quartiles	1°	0.072	0.035	0.086	0.163	0.083	0.136	0.136	0.126	0.072	0.094	0.166	0.126	0.126	0.215	0.2	0.09
			2°	0.082	0.072	0.109	0.208	0.152	0.213	0.213	0.187	0.146	0.21	0.224	0.2	0.221	0.253	0.23	0.221
			3°	0.139	0.157	0.16	0.259	0.199	0.245	0.312	0.221	0.213	0.221	0.254	0.326	0.368	0.293	0.293	0.301

	Multivariate linear regression *p *values to predict weight-adjusted TAC dose (mg/kg/day)																		
	Day 1	Day 2	Day 3	Day 4	Day 5	Day 6	Day 7	Day 8	Day 9	Day 10	Day 11	Day 12	Day 13	Day 14	Day 15				
GRWR (Unstand. B coeff.)	0.401 (-)	0.714 (-)	0.089 (0.012)	0.161 (-)	0.003 (0.024)	0.078 (0.017)	0.124 (-)	0.224 (-)	0.065 (0.021)	0.006 (0.037)	0.008 (0.03)	0.032 (0.024)	0.006 (0.037)	0.046 (0.023)	0.009 (0.039)				
Donor CYP3A5 genotype (Unstand. B coeff.)	0.092 (− 0.049)	0.004 (− 0.089)	< .001 (− 0.086)	< .001(− 0.078)	< .001 (− 0.099)	0.106 (-)	0.047 (− 0.064)	0.125 (-)	0.012 (− 0.090)	0.369 (-)	0.17 (-)	0.018 (− 0.094)	0.01 (− 0.115)	0.005 (− 0.117)	0.05 (− 0.079)				
Determ. Coeff. (R^2^)	0.093	0.194	0.256	0.31	0.336	0.091	0.126	0.064	0.157	0.139	0.119	0.183	0.239	0.253	0.172				
F statistic (d.f. , res.)	4.22 (1, 47)	10.1 (1, 47)	14.41 (1, 47)	18.89 (1, 47)	10,10 (2, 46)	2.59 (2, 46)	3.16 (2, 46)	1.51 (2, 46)	4.09 (2, 46)	7.24 (1, 47)	6.08 (1, 47)	4.26 (2, 46)	5.97 (2, 46)	5.59 (2, 46)	6.44 (1, 47)				
Intercept	0.194	0.268	0.242	0.261	0.236	0.084	0.23	0.168	0.279	0.052	0.07	0.255	0.254	0.334	0.059				

### Clinical significance

Among the enrolled patients, 20 out of 49 (41%) patients developed acute rejection during the first 15 post-transplant days, at a median of 9 post-transplant days (range 7–30). None of these events led to graft failure or patient death.

Genetic, anthropometric or demographic variables were not significantly associated with the onset of acute rejection.Only ALT levels on the 1st post-transplant day were significantly higher in patients who developed acute rejection in the following days. The protocol assumption of corticosteroids as induction immunosuppression did not prevent the onset of acute rejection.

Patients with acute rejection had a lower TAC C/D/Kg than patients with no rejection, even if this did not reach statistical significance. This trend was present from the first to the 10th post-transplant day, followed by a reversed trend. Acute rejection was not significantly associated with recipient or donor genotype, age or gender. Noteworthy was the fact that acute rejection occurred with statistical significance more frequently in the group of patients who received an extra-large graft (GRWR > 5%). The logistic regression analysis evidenced that only the GRWR had a statistically significant predictive value for the occurrence of acute rejection (*p *value = 0.005). Patients with a GRWR > 4% had a higher risk of acute rejection than those with a ratio < 4% (AUROC 0.742, *p *value = 0.004), with a 70% sensitivity and an 82% specificity (Positive Predictive Value = 73.7% and a Negative Predictive Value = 80%).

Incidences of early post-transplant surgical complications such as hepatic artery thrombosis, portal vein thrombosis, biliary complications, intra-abdominal bleeding or intestinal perforation were all comparable in our cohort regardless the GRWR. For the entire cohort, 1-, 3-, 5-year patient and graft survival rates after liver transplant were 95.8%. Patient and graft survival did not differ among the three groups of patients stratified by GRWR: 1-, 3-, and 5-year patient and graft survival rates were 95.2%, 95.0%, and 100% respectively, for children who received size-matched, large and extra-large grafts.

## Discussion

Currently, immunosuppressive treatment with calcineurin inhibitors requires continuous monitoring and dose adjustments, particularly in the first two weeks of treatment, in order to avoid graft rejection or treatment toxicity. This optimization goal is currently achieved through the adoption of a routine TDM. In our study, despite the effectiveness of the TDM in gradually optimizing the posology, many patients had TAC concentrations out of the therapeutic ranges during the first days. This appeared to be due to inadequate TAC starting dose, which is usually determined based on patients’ weight.

The TAC pharmacokinetic analysis we performed in the first two post-transplant weeks confirmed some results from our previous study^12^, where the donor *CYP3A5* genotype and the recipient age explained part of the inter-individual variability in TAC concentrations. In the present study, we confirmed that patients receiving a liver from donors heterozygous for the CYP3A5*3 allele have higher dose requirements compared to those who are homozygous for the CYP3A5*3 allele, as previously reported^8–11^, suggesting that the hepatic metabolism is determinant on TAC blood concentrations immediately after the transplant, regardless of the impaired hepatic synthetic function, which characterizes the early post-transplant period. On the other hand, the recipient *CYP3A5* resulted not significantly associated with TAC pharmacokinetics, suggesting that the extrahepatic metabolism may have a limited impact, even in the first few days. On the contrary, *CYP3A4* genotype showed no significant impact on TAC metabolism in our cohort, probably due to the low sample size (the stronger effect of *CYP3A5* could mask factors with lower impact). However, although a minor role of *CYP3A4* polymorphisms on TAC pharmacokinetics has been recently reported in the consensus report on TAC personalized therapy^2^, data are more controversial in pediatric liver transplant patients, whereas it may be more relevant in kidney transplant patients^23–25^.

Moreover, although P-glycoprotein (encoded by *ABCB1* gene) may have a role in TAC disposition (particularly in lymphocytes^29,30^), we did not observe any significant association with dose requirements, in line with previous reports^26–28^.

We confirmed the impact of the recipient age, suggesting that children under 8 years of age should be given higher TAC doses, particularly if they are under 2, whereas metabolic pathways in patients over 8 years of age may be more similar to adults^31–33^.

Nevertheless, when the pharmacokinetic analysis was extended to cover the first two post-transplant weeks, we observed that the impact of the aforementioned factors had on TAC pharmacokinetics changed during the first post-transplant days and most likely reflected changes in the patient’s physiology and pharmacokinetics. Indeed, the patients’ gender and age had no significant effect on the TAC exposure, whereas an increase in the influence of GRWR on TAC blood concentrations was observed. The recipient age could play an important role in determining TAC concentrations during the first two post-transplant days, probably due to differences in the volume of distribution, whereas the graft size could become a better predictive factor of TAC concentrations later. According to this hypothesis, younger recipients might require higher TAC doses since they generally receive relatively larger livers. As already reported, the standard liver volume to body weight ratio (as well as GRWR) of children decreases as age increases until approximately the age of 16 years old^34^, therefore GRWR could explain the age-related variability in TAC pharmacokinetics. Conversely, our results showed that the type of transplant graft (whole/reduced/split liver) had no relevant influence on TAC blood levels, as previously reported^35,36^.

Our evidence about the impact of GRWR on TAC metabolism is in line with a recent study on a large Japanese cohort^19^, and suggests that patients who receive a larger graft, due to their higher amount of hepatic tissue (and hence enzymes), have enhanced TAC metabolism. This evidence is theoretically quite reasonable, so much that graft weight is a key variable for the calculation of the metabolic capability of individuals, and it is considered as a main feature for the physiologically based pharmacokinetic (PBPK) modeling^37^. Therefore, a GRWR-based TAC initial dose could be more accurate than the current standard weight-based dose.

Nevertheless, probably due to the low sample size (and so low statistical power), the significant correlation between GRWR and TAC C/D/Kg was observed up to the 10th post-transplant day (and then it lost of significance), whereas in the Japanese study it occurred from 6 to 12 months after transplant^19^. Other factors could contribute to this difference with the Japanese study. The study by Shoji et al*.*^19^ was focused on transplants from living donors, while our patients received graft from deceased donors. The median graft size was larger in our study (GRWR 3.3% vs 2.37%) and a different distribution in GRWR groups was observed, e.g. no small-for-sizegrafts were observed in our population.

On the other hand, the regeneration of the transplanted liver may be relatively fast in the first post-transplant weeks, as already reported previously^38–42^: this may occur very early in the first post-transplant weeks, with 80% of volumetric recovery after 1 week from transplant^38^ and graft size normalization within the 2nd post-transplant month. Moreover, in our sample, we observed higher ALT levels, underlying more cytolysis, and probably a consequent reduction in the amount of functional liver tissue, in patients with higher GRWR. These concomitant mechanisms, e.g. liver regeneration and cytolysis, may progressively reduce the differences in TAC metabolism between small-for-size and large or extra-large-for-size grafts: the interindividual variability in GRWR would rapidly decrease within the first weeks according to the initial graft size, reaching a very low variability after two months, thus explaining both our results and those by Shoji et al.^19^.

Most important, in our cohort the vast majority of acute rejections and TDM-guided dose adjustments were applied around the 9th day of treatment: acute rejection can slightly decrease the hepatic metabolism of TAC (this was not statistically significant, but a reversion in the trend was observed compared with the previous days), leading to an increase in the TAC C/D/Kg in patients with rejection. Interestingly, acute rejection was more frequent in patients with high GRWR, so this phenomenon would also explain the change in the predictive power of GRWR for TAC C/D/Kg.

Finally, the dose adjustments applied (particularly around the 9th post-transplant day), based on TDM results and/or on the occurrence of acute rejection, may also cause a slight bias in the C/D/Kg; in fact, the observed trough TAC blood levels in the following days cannot be considered as the steady-state concentrations, causing fluctuations in the ratio until a new steady-state is reached.

Therefore, the most reliable data in terms of prediction of C/D/Kg are surely the ones within the 9th post-transplant day, before the introduction of these sources of bias.

Safety criteria of GRWR for pediatric patients are greatly influenced by the age of recipients and still represent a matter of intense debate, especially for infant recipients, where the impact of graft-to-recipient size mismatching on outcomes remains unclear. Liver transplant techniques using living-related grafts, reduced-size livers, and split livers have been widely exploited due to the shortage of size-matched cadaveric organs. Nevertheless, the reasonable range of GRWR in infants has not been well defined. The reduction in the graft mass may be an adequate strategy in selected cases to limit collateral risks of oversizing, but it should not be proposed systematically as it may incur an added risk of complications caused by greater parenchymal surface and suboptimal vascular flow. In our series, the high median GRWR was dependent on the surgical decision to prefer whole liver transplants in children < 2 years old and to eventually use temporary tension-free abdominal closure in cases of increased abdominal pressure. Our data showed that all the patients who received a large o extra-large graft achieved excellent post-transplant survival outcomes, suggesting that the accumulation of technical experience is relevant for the successful implant of large/extra-large grafts in infants. Nevertheless, our personal experience with this research showed that the onset of acute rejection was significantly associated with the patient’s GRWR, meaning that patients who have a larger than proportional graft size also run a higher risk of developing rejection, i.e. we observed that a GRWR cut-off value of > 4% was strongly predictive of acute rejection. This observation is in line with those of Kiuchi et al., who found that acute rejection was more frequent in larger-for-sizegrafts^18^. Other authors observed that acute rejection rate was not associated to GRWR^43^, however, these different findings could be related to the adoption of different induction immunosuppression protocols.

Acute rejection was not significantly associated to other donor/recipient and graft characteristics, but it occurred more frequently in patients with lower TAC blood concentrations, even if this association did not reach statistical significance, probably due to the small sample size. This indicated that acute rejection might depend on the size of the graft, not only because there is more tissue capable of metabolizing the TAC, leading to lower systemic concentrations, but probably also because an extra-large sized graft means more allo-antigens and a more extended tissue damage, as sustained by the correlation between ALT levels and the GRWR on the 1st post-transplant day. Moreover, a larger liver could mean also a less homogeneous drug penetration in the first days after transplantation, so it could take longer to achieve therapeutic levels in the whole graft. Nevertheless, the surgical decision to use large or extra-large grafts should not be limited on the basis of our results, if it is supported by a valid technical experience. On the other hand, we believe that the TAC starting dose should be increased when a pediatric liver transplant is performed by using a proportionally large or extra-large graft.

The multivariate regression analysis suggests that the best predictors of the final TAC dose needed to reach therapeutic concentrations are GRWR and the donor *CYP3A5* genotype. Therefore, the TAC starting dose could be successfully guided and individualized according to the donor metabolic activity and graft size, allowing to achieve the TAC concentrations in the therapeutic in a shorter time and reduce the risk of acute rejection.

**Figure 3:**
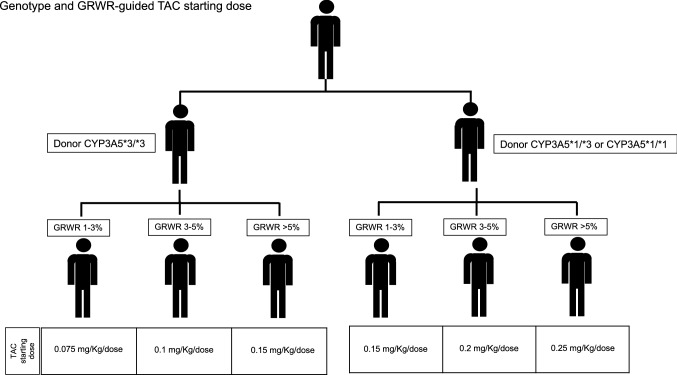
Proposal for an algorithm for a genotype and GRWR guided TAC starting dose. TAC, tacrolimus; GRWR, Graft-to-Recipient Weight Ratio.

Our findings suggest that it may be of advantage stratifying genotype-guided dosing by the graft size (GRWR) instead of the recipient age^17^. We reported in Fig. 3 a proposal for an algorithm for individualized TAC starting dose on the basis of our results, including the donor *CYP3A5* and the GRWR, where the recommended doses need to be validated in larger sample sizes. This hypothesis might lay the groundwork for a future prospective trial that would allow implementation of an efficacious guided dosing for the TAC starting dose and the subsequent dose titrations in the early post-transplant period.

Prior studies in adults reported a higher risk of rejection, poor graft outcomes and longer hospital stay in patients with sub-therapeutic or supra-therapeutic concentrations compared to those with therapeutic concentrations^44–46^. Confirmation of whether a more precise individualized dosing has a potential to improve patients’clinical outcomes and reduce hospital length of stay and costs associated with complications is to be obtained through further research.

In conclusion, defining the donor genetic profile (*CYP3A5* genotype) and the graft size (GRWR) of the liver transplant before starting the TAC–based immunosuppression may be an attractive option for the prediction of the individual dose required and could well allow for a quicker achievement of target blood concentrations. We confirmed that the testing of the donor *CYP3A5* genotype should be routinely implemented by clinical pharmacology and pharmacogenetics laboratories for all children. Although the donor *CYP3A5* genotype seems to be the most relevant predictive factor on TAC pharmacokinetics, we suggest that GRWR, which is readily available to clinicians at the time of transplant, could be used not only for its surgical impact, but also as a predictive factor to guide the TAC starting dose.

The findings of this study should be an incentive for developing TAC dosing algorithms, incorporating clinical and genetic factors, in order to minimize the inter-individual variability in TAC concentrations and potentially reduce post-transplant complications. Multicenter large-scale studies are required to more comprehensively explore the impact of the use of large grafts on TAC pharmacokinetics and the risk of acute rejection in infants < 5 kg.

## Abbreviations

TAC: Tacrolimus
TDM: Therapeutic drug monitoring
GRWR: Graft-to-Recipient Weight Ratio
C/D/Kg: Concentration/weight-adjusted dose ratio
